# Quantitative *in vivo* optical tomography of cancer progression & vasculature development in adult zebrafish

**DOI:** 10.18632/oncotarget.9756

**Published:** 2016-06-01

**Authors:** Sunil Kumar, Nicola Lockwood, Marie-Christine Ramel, Teresa Correia, Matthew Ellis, Yuriy Alexandrov, Natalie Andrews, Rachel Patel, Laurence Bugeon, Margaret J. Dallman, Sebastian Brandner, Simon Arridge, Matilda Katan, James McGinty, Paul Frankel, Paul M.W. French

**Affiliations:** ^1^ Department of Physics, Imperial College London, London SW7 2AZ, UK; ^2^ Division of Medicine, University College London, London WC1E 6JF, UK; ^3^ CoMPLEX, University College London, London WC1E 6BT, UK; ^4^ Department of Life Sciences, Imperial College London, London SW7 2AZ, UK; ^5^ Department of Computer Science, University College London, London WC1E 6BT, UK; ^6^ Department of Neurodegenerative Disease, UCL Institute of Neurology, London WC1N 3BG, UK; ^7^ Institute of Chemical Biology, Department of Chemistry, Imperial College, London SW7 2AZ, UK; ^8^ Division of Neuropathology, The National Hospital for Neurology and Neurosurgery, University College London NHS Foundation Trust, London WC1N 3BG, UK; ^9^ Division of Structural and Molecular Biology, University College London, London WC1E 6BT, UK

**Keywords:** cancer, adult zebrafish, optical projection tomography, hepatocellular carcinoma, KRas

## Abstract

We describe a novel approach to study tumour progression and vasculature development *in vivo* via global 3-D fluorescence imaging of live non-pigmented adult zebrafish utilising angularly multiplexed optical projection tomography with compressive sensing (CS-OPT). This “mesoscopic” imaging method bridges a gap between established ~μm resolution 3-D fluorescence microscopy techniques and ~mm-resolved whole body planar imaging and diffuse tomography. Implementing angular multiplexing with CS-OPT, we demonstrate the *in vivo* global imaging of an inducible fluorescently labelled genetic model of liver cancer in adult non-pigmented zebrafish that also present fluorescently labelled vasculature. In this disease model, addition of a chemical inducer (doxycycline) drives expression of eGFP tagged oncogenic K-RASV12 in the liver of immune competent animals. We show that our novel *in vivo* global imaging methodology enables non-invasive quantitative imaging of the development of tumour and vasculature throughout the progression of the disease, which we have validated against established methods of pathology including immunohistochemistry. We have also demonstrated its potential for longitudinal imaging through a study of vascular development in the same zebrafish from early embryo to adulthood. We believe that this instrument, together with its associated analysis and data management tools, constitute a new platform for *in vivo* cancer studies and drug discovery in zebrafish disease models.

## INTRODUCTION

There is increasing interest in biomedical research and drug discovery to study biological processes *in situ* in live organisms utilising whole-body molecular imaging [1]. Fluorescence imaging can provide *in vivo* molecular contrast [2] and established 3-D microscopy techniques such as laser scanning confocal or multiphoton fluorescence microscopy can provide subcellular resolution imaging. However, the image acquisition times can become prohibitively long for whole-body imaging of larger (>1 mm) scale samples and the extended exposure to focussed excitation light can lead to photobleaching and to phototoxicity in live subjects. For most animal models, *in vivo* imaging is further compromised by strong absorption and scattering of optical radiation and there is a dearth of approaches to bridge the “gap” between optical microscopy of small (~mm scale) organisms and global imaging of murine disease models that is currently implemented [3] using “planar imaging” techniques or diffuse tomography. This “gap” can be addressed by hybrid photoacoustic techniques, e.g. [4,5] but these are not yet able to access the full range of readouts available to fluorescence imaging.

For transparent samples, a variety of 3-D mesoscopic fluorescence imaging techniques have been developed for both *ex vivo* and *in vivo* studies, including optical projection tomography (OPT) [6,7], scanning laser optical tomography (SLOT) [8] and light sheet microscopy (LSM) [9], These techniques can form diffraction-limited, 3-D images of transparent mesoscopic samples at higher frame rates and much reduced photobleaching/phototoxicity compared to laser scanning microscopy. They have been successfully applied to fixed samples that have been chemically cleared and to live, partly transparent, organisms such as *Drosophila* [10], *C. Elegans* [11] and early stage zebrafish (*Danio rerio*) embryos [12,13]. OPT provides the advantages of low phototoxicity associated with the wide-field illumination, simplicity, relatively low cost of implementation (requiring only sample rotation) and ease of scaling the imaging system to larger samples. It also offers the opportunity to reduce light dose and acquisition time using compressive sensing (CS) [14] by taking advantage of iterative reconstruction algorithms, previously developed for x-ray computed tomography, that permit 3-D images to be reconstructed with many fewer angular projections than required for fully sampled filtered back-projection (FBP).

In support of translational oncology research, we have developed an enabling new imaging modality to study tumour progression and vasculature development *in vivo* using global 3-D fluorescence imaging of live non-pigmented adult zebrafish. Due to their rapid generation time and genetic accessibility, zebrafish are increasingly used to model disease and aid drug development [15]. To date, most imaging studies have focused on the use of optically clear embryos, which can be used to monitor organ development and differentiation. However, the use of embryos has considerable limitations for many diseases settings where development of the animal is key such as the requirement for a mature vasculature and immune system in cancer. Recently, adult zebrafish have been used to faithfully model tumour progression, with significant similarities to human disease progression [16,17,18]. While wild-type adult zebrafish are not amenable to optical imaging, there are now non-pigmented mutants available of which the adults are sufficiently transparent to permit optical readouts [19,20,14]. Here we describe the development and application of angularly multiplexed, compressive sensing optical projection tomography (CS-OPT) for 3-D global fluorescence imaging of liver cancer in adult TraNac zebrafish, enabling *in vivo* and longitudinal quantitation of tumour progression and vascular development. To illustrate its potential, we have applied this instrument to image changes in tumour and vasculature associated with hepatocellular carcinoma (HCC) in live tumour burdened adult transgenic (Tg) TraNac zebrafish, TraNac *Tg (KDR:mCherry:Fabp10-rtTA:TRE-eGFPKRAS^V12^*)**. These zebrafish are immune competent and display mCherry expression in the vasculature and hepatocyte specific expression of eGFPKRAS^V12^ upon treatment with doxycycline (DOX). Continued treatment leads to liver-specific eGFP-labelled tumorigenesis with an HCC phenotype, which is reversible upon withdrawal of DOX [21].

## RESULTS AND DISCUSSION

**Figure 1:**
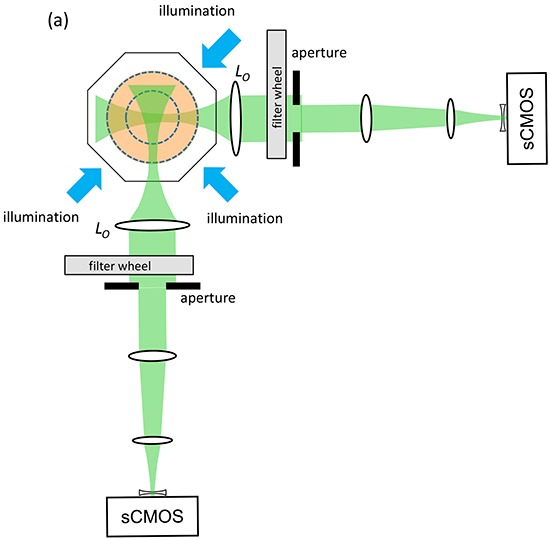
Schematic of dual projection channel multispectral OPT system

Figure 1 shows the configuration of the novel OPT instrument we have designed for 3-D global imaging of anaesthetised adult zebrafish. To increase light collection efficiency and spatial resolution, we have implemented multiplexed OPT with two imaging arms of approximately 0.5x magnification focussed to different planes within the sample [22] that acquire projection images with a diffraction-limited resolution of 26 μm. As described in the Materials and Methods section, this has been configured to acquire sets of angularly resolved projection images in spectral channels optimised for eGFP and mCherry fluorescence. To facilitate the analysis, management and remote access of the large multispectral 3-D data sets, we have developed an OPT data workflow that utilises OMERO [23] (Supplementary Figure S2).

In initial studies to optimise our imaging protocols, we determined that starved male, homozygous KDR:mCherry^+/+^ fish should be used (Supplementary Figure S3) and, in order to minimise the duration of the imaging procedure, we undertook an investigation of the trade-off between the reconstructed 3-D image quality and the number of angular projections acquired. This was undertaken by imaging an adult tumour-burdened zebrafish after 3 weeks' treatment with DOX and acquiring 512 angular projections. We then applied the structural similarity index measure (SSIM) to segmented 3-D image reconstructions obtained from subsets of the acquired image data with varying numbers of projections and compared the results to that obtained from the full 512 projection FBP reconstruction, which was used to represent the “ground truth”. This preliminary study indicated that reconstructing with 64 projections using the “compressive sensing OPT” (“CS-OPT”) approach [14] was optimal for image reconstruction (as indicated in Supplementary Figure S4 and S5).

**Figure 2:**
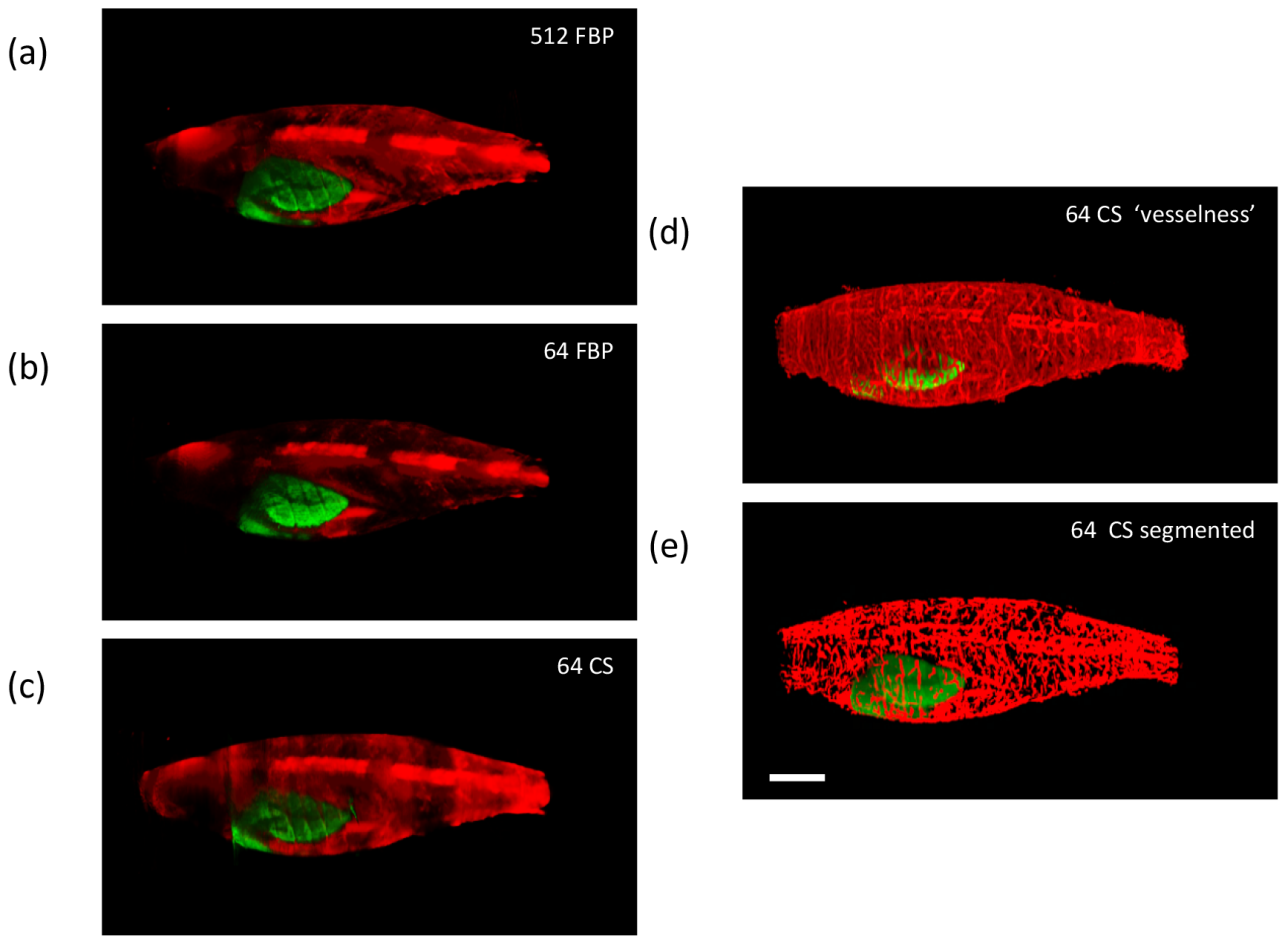
Exemplar reconstructed OPT images of adult [81 days post fertilization (dpf)] TraNac Tg (*KDR:m Cherry:Fabp10-rtTA:TRE-eGFPKRASV^12^*) zebrafish expressing liver specific eGFP-labelled tumour and mCherry-labelled vasculature showing maximum intensity projections of eGFP (green) and mCherry (red) fluorescence (scale bar = 2.5 mm) **a.** and **b.** show FBP reconstructed images computed with 512 and 64 projections respectively, **c.** shows the corresponding image reconstructed from 64 projections using the CS-OPT approach, **d.** shows the “vesselness” of the same reconstruction and **e.** shows the “final” segmented image of the vasculature. See Supplementary Video S1 for corresponding 3-D animations.

Figure 2a shows the fluorescence intensity image of this adult tumour-burdened zebrafish reconstructed from a 512 projection FBP and Figure 2b shows how this image is degraded if only 64 projections are used in a FBP reconstruction. Figure 2c shows the fluorescence intensity image reconstructed from the same 64 projections using the iterative CS algorithm. Following reconstruction, the 3-D image stacks were segmented and skeletonized to produce binary images of the vasculature, as discussed in the Materials and Methods section. Figure 2d shows the “vesselness” of this 3-D dataset visualised after Hessian-based analysis using MATLAB and Figure 2e shows the “final” segmented image following skeletonization and dilation of the vasculature. Rendered 3-D images of these corresponding figures are shown in Supplementary Video S1. In general, this CS-OPT approach exploiting under-sampling and iterative tomographic reconstruction is attractive for *in vivo* imaging since it reduces the light dose and the time that the animal must be maintained under anaesthetic.

**Figure 3:**
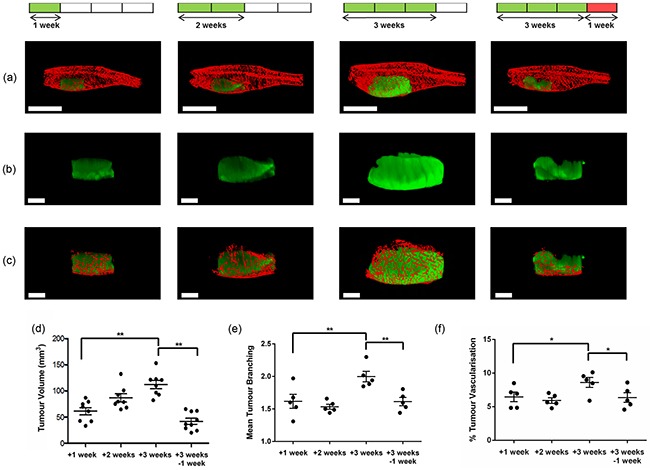
**a-c.** Representative “final” segmented reconstructed CS-OPT images of eGFP-labelled tumour and mCherry-labelled vasculature in adult TraNac *Tg (KDR:mCherry;Fabp10:rtTA;TRE:eGFP:KRAS^V12^*)** zebrafish with data acquired following DOX treatment at 145 dpf after one, two and three weeks of treatment and after three weeks of DOX treatment plus a further week with no treatment (See also Supplementary Videos S2–S3). (a) shows whole images of tumour and vasculature (scale bar = 5 mm); (b) and (c) show the corresponding CS-OPT images (scale bar = 2 mm) of the of the tumour alone and of the segmented vasculature associated with the tumour. **d, e, f.** respectively are graphs plotting the corresponding variation of the mean tumour volume, the mean number of tumour vessel branch points and percent tumour vascularisation (proportion of vasculature volume within tumour volume) calculated from this OPT data set, for which n≥5 at each condition and the error bars represent the standard error of the mean (SEM). *p<0.05, **p<0.01 as indicated by lines.

Having optimised the conditions for acquisition of adult tumour burdened zebrafish using CS-OPT, we performed an *in vivo* cross-sectional study of tumour and vasculature development following treatment with - and withdrawal of - DOX. Adult TraNac *Tg (KDR:mCherry: Fabp10-rtTA:TRE-eGFPKRAS^V12^)* zebrafish were treated with DOX and imaged after 1, 2 and 3 weeks of treatment and after a 4^th^ week of no treatment. Continued treatment has been reported to lead to a hyperplastic liver phenotype associated with progression to HCC, which is reversible upon withdrawal of DOX due to the oncogene addicted nature of the model resulting in rapid clearance of the tumour through apoptosis [17]. The maximum intensity projection (MIP) reconstructions of Figure 3a-3c (and Supplementary Videos S2–S3) show the qualitative changes in induced eGFP-labelled tumour and the mCherry-labelled vasculature during progression. Quantitative analysis of the reconstructed 3-D images was undertaken and Figure 3d shows the significant increase in tumour volume observed between 1 and 3 weeks DOX treatment and the significant decrease between 3 weeks treatment and 1 week DOX withdrawal. Quantitative analyses of the tumour vasculature based on segmented 3-D imaged datasets (Figure 3e, 3f and Supplementary Figure 6), show increases in branching and percent tumour vascularisation consistent with HCC tumour angiogenesis [24], and which is reversed upon DOX withdrawal. We also analysed the mean vessel diameter and length and found no significant differences (Supplementary Figure S6).

**Figure 4:**
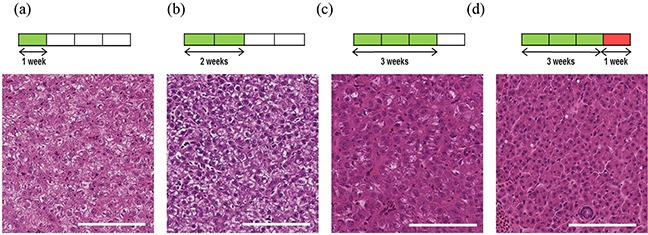
Induction and progression of HCC in adult TraNac *Tg* (*KDR:mCherry:Fabp10-rtTA:TRE-eGFPKRAS^V12^*) zebrafish Images of H&E stained representative tumour sections following tumour induction for **a.** one week, **b.** two weeks and **c.** three weeks with **d.** imaged after three weeks of induction plus a further week after removal of inducer treatment. Each group (n=6-8) Scale bars 100 μm.

**Figure 5:**
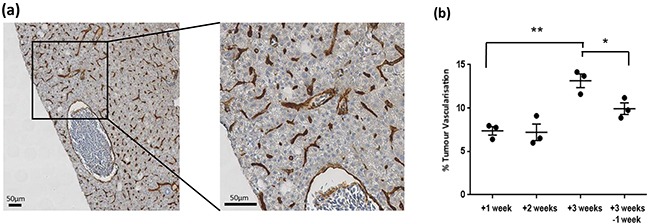
**a.** Representative IHC mCherry staining showing vessels (brown) from three week tumour induced adult TraNac *Tg* (*KDR:mCherry:Fabp10-rtTA:TRE-eGFPKRAS^V12^*) zebrafish. Scale bar 50μm. **b.** Graph showing IHC mCherry quantitative analysis of percent vessel coverage over regions of interest. Each group (n =3) and error bars represent the SEM. *p<0.05, **p<0.01 as indicated by lines.

After imaging, tumours were removed and subjected to histological analysis for comparison to CS-OPT data. Using H&E staining we confirmed that DOX treatment leads to progression towards an HCC phenotype as previously described [17] (Figure 4). In addition, quantitative analysis of the percentage tumour vascularisation using immunohistochemistry (IHC) with an mCherry antibody (Figure 5a) in regions corresponding to segmented vasculature in the CS-OPT data revealed similar levels and trends in vessel coverage as can be seen by comparing Figure 5b and Figure 3f.

This agreement in tumour vascularisation is encouraging because CS-OPT is rapid and non-invasive and the analysis can be applied to the whole tumour –or the whole fish. However, it should be noted that CS-OPT does not account for scattering or aberrations of the emitted fluorescence and the imaging performance is reduced towards the centre of adult zebrafish. Thus, as shown in Supplementary Figure 7 and Supplementary Video S3, the ability of the CS-OPT image reconstruction and subsequent image segmentation to visualise the tumour vasculature can vary throughout individual fish due to variations in their optical properties. While this instrument does already provide a useful platform for studying cancer progression, its capabilities could be improved through the use of red and/or infrared fluorophores, since the optical scattering would be reduced at longer wavelengths. The imaging platform could be further developed to incorporate more advanced algorithms for tomographic reconstruction using weakly scattered light or by implementing techniques for selecting ballistic (non-scattered) light.

**Figure 6:**
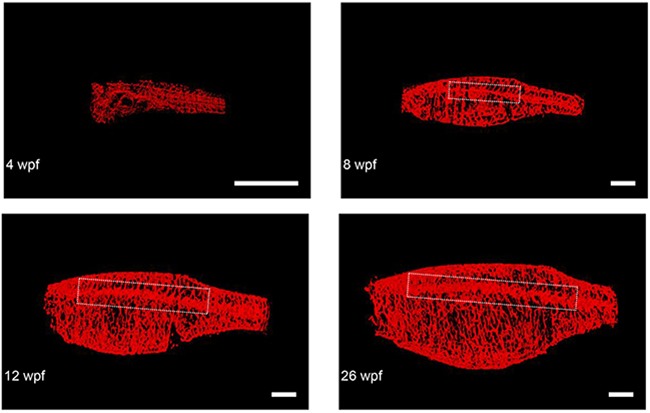
Representative final reconstructed CS-OPT images from a longitudinal study of an individual TraNac Tg (*KDR:mCherry*) zebrafish re-imaged at 4, 8, 12 and 26 weeks post fertilisation (wpf) Inset boxes define the vascular region containing the lateral cutaneous artery and Table 1 summarises the quantification of key parameters of this developmental angiogenesis. (scale bar = 2 mm) (See also Supplementary Video S4).

To demonstrate how the minimally invasive nature of this *in vivo* CS-OPT platform, coupled with the rapid acquisition time, can enable longitudinal studies of individual zebrafish, we repeatedly imaged an individual TraNac Tg(KDR:mCherry) zebrafish over a 26 week period. The results are illustrated in Figure 6, which shows maximum intensity projections of segmented 3-D reconstructions of the mCherry-labelled vasculature of an individual TraNac *Tg(KDR:mCherry*) zebrafish imaged repeatedly over a 26 week period and Supplementary Video S4 shows the corresponding 3-D renderings. Quantitative analysis of developmental angiogenesis of the lateral cutaneous artery shows increases in vascular length, diameter, and branching, as summarised in Table 1.

**Table 1:** Quantification of developmental angiogenesis of the lateral cutaneous artery in a TraNac Tg (KDR:mCherry) zebrafish longitudinally imaged using CS-OPT at 8,12 and 26 weeks post fertilisation (wpf)

	8 wpf	12 wpf	26 wpf
Length (mm)	7.09	11.00	16.01
Mean Diameter (μm)	159.82	253.22	265.24
Number of Branchpoints	36	57	65

In conclusion, we present a novel 3-D imaging platform based on angularly multiplexed OPT with compressive sensing that has been optimised for imaging live tumour burdened adult zebrafish. Using only 64 angular projections, the total data acquisition time for both tumour and vascular channels was less than 3.5 minutes, which is important for zebrafish viability and application to longitudinal studies. We have shown that CS-OPT can be applied to image the progression of HCC in adult zebrafish and we have demonstrated the capability of this platform for longitudinal imaging studies of adult zebrafish. We believe this work indicates the potential of CS-OPT to provide robust quantitative measurements of disease development and to evaluate anti-cancer therapies. This capability can be implemented at a cost that is comparable to a wide-field fluorescence microscope.

## MATERIALS AND METHODS

### Transgenic zebrafish

The transgenic lines used within the study were TraNac *Tg*(*KDR:mCherry:Fabp10-rtTA:TRE-eGFPKRAS^V12^*) and TraNac *Tg*(*KDR*:*mCherry*). These lines were generated through the breeding of transparent TraNac zebrafish (gift from Julian Lewis, Cancer Research Institute), a double mutant for the *mitfa/nacre* and the *mpv17/transparent* genes, to both *Tg(KDR:mCherry)* fish (gift from Steve Wilson, University College London) and *Tg*(*Fabp10-rtTA:TRE-eGFPKRAS^V12^*) fish (gift from Zhiyuan Gong, National University of Singapore). The resultant TraNac *Tg(KDR:mCherry)* and TraNac *Tg* (*Fabp10-rtTA:TRE-eGFPKRAS^V12^*) were crossed to generate the transparent line with mCherry labelled vasculature and an inducible, liver-specific GFP labelled tumour.

### Zebrafish husbandry

Zebrafish lines were bred and maintained within the UCL fish facility and the Central Biomedical Services facility at Imperial College London according to standard practices where all procedures conformed to UK Home Office requirements (PPL 70/7700 & PPL 70/8365). Breeding and maintenance were carried out in system water, with the addition of 0.0003% (w/v) methylene blue to embryos. Tumour induction was performed through the addition of doxycycline (Sigma D-9891) in fish water at a concentration of 10mg/L. For imaging, male zebrafish, starved for 24 hours, were anaesthetised with 4.2% Tricaine solution before acquisition. After which the fish were transferred to a recovery tank, monitored to regain consciousness, before being returned to storage tanks.

### Imaging instrumentation

The angularly multiplexed [22] two channel OPT instrument represented in Figure 1 was designed to accommodate adult zebrafish up to 5 cm in length and 1 cm in diameter and provide a field of view of 33.8 × 28.5 mm. In each channel the optical imaging system depicted in Supplementary Figure 1 provided a magnification of 0.49x with images acquired using sCMOS cameras (Zyla 5.5 sCMOS, Andor Technology Ltd) with an imaging sensor of 16.6 × 14.0 mm with 2560 × 2160 pixels of 6.5 μm. The effective numerical aperture (NA) of each imaging arm was adjusted using the adjustable iris in the back focal plane of the “objective” lenses L_1_ to provide a depth of field of 2.5 mm with a diffraction limited point spread function of FWHM 18-22 μm depending on the wavelength. This is close to the Nyquist limit of 26 μm determined by the system magnification and the pixel size of the camera. One imaging arm was focused to 1.25 mm and the other to 3.75 mm from the axis of rotation. These imaging arms were optimised using WinLens (www.winlens.de) for minimum aberrations and field curvature across the field of view.

### Image acquisition

For imaging, anaethetised fish were transferred into a clear tube of 9.5 mm internal diameter (11 mm outside diameter) made of FEP (fluorinated ethylene propylene) tubing with a refractive index of 1.34 (EW-06406-12, Cole Parmer Instrument Co Ltd) with care taken to minimise any bubbles within the tube. These tubes were then sealed with a plug and mounted under a rotation stepper motor (T-NM17A200, Zaber Technologies Inc.) set to run using a sufficiently low level of acceleration to prevent ‘slipping’ of the fish within the tube. Typically, the acceleration used was 10 microsteps/s^2^ for a motor with 12800 microsteps/revolution (T-NM17A200), corresponding to an angular acceleration of ~1.6 deg/s^2^. The tube was suspended in a custom-built imaging chamber of octagonal cross-section filled with water to provide index matching of the tube and with glass windows normal to the imaging and excitation axes. This chamber allowed for the sample to be illuminated from multiple directions simultaneously in order to improve uniformity of fluorescence excitation. The collimated excitation laser beams were directed onto a rotating engineered diffuser (ED1-C50-MD, Thorlabs, Inc) with a cone angle of 50 degrees and the resulting scattered light was collimated with a Fresnel lens of 50.8 mm diameter (FRP251, Thorlabs, Inc) and 51 mm focal length. Because adult zebrafish typically present an aspect ratio >4:1 (i.e. >4 cm length and ~1 cm diameter), it is possible to more efficiently utilise this excitation light by dividing it into three sub-beams with similar aspect ratio to the fish. The OPT image projection data were acquired by the two sCMOS cameras running in parallel interfaced with separate computers and the acquisition process was controlled by custom software written in LabVIEW (National Instruments) such that one computer was slaved to the other, which also controlled the rotation stage motor, and the projection images were acquired in synchronism. Multispectral imaging of the eGFP-labelled tumour and the mCherry-labelled vasculature was implemented by acquiring two data sets sequentially with excitation at 488 nm and 561 nm, for which exposure times of 1 s and 2 s respectively were typically required for each projection at the maximum excitation power levels of up to 150 mW at 488 nm provided by a diode laser (MLD, Cobalt AB) and up to 200 mW at 561 nm provided by a diode-pumped solid-state laser (Jive, Cobolt AB).

### Data management

Following the data acquisition, each series of angular projections was manipulated computationally to ensure that the rotation axis was centred in the adjusted dataset and the two multiplexed camera datasets were then co-registered using a calibration process incorporating magnification, translation and rotation with rigid body transforms. The co-registered angular projections were then saved as OME-TIFF files on an OMERO [23] server to enable sharing of these large datasets with remote access across the internet. This angular projection dataset requires much less storage capacity than the 3-D reconstructed image stacks. The projection data can subsequently be downloaded and the 3-D image stacks reconstructed on demand. Supplementary Figure 2 presents an overview of the data workflow.

### Image reconstruction

OPT datasets comprising co-registered sets of angular projections from each sCMOS camera saved as OME-TIFF files could be directly reconstructed using Filtered Back Projection (FBP) via a MATLAB (The MathWorks Inc) programme written in-house using the standard iradon library function. FBP reconstruction ideally requires several 100 angular projections to reconstruct 3-D images without loss of information using the imaging system reported here. When implemented on a personal computer with a graphics processing unit (NVIDIA Tesla K40 GPU), this typically takes a few minutes for a data set with 200 projections that reconstructs to a z-stack of 2000 image planes.

The CS-OPT approach is applicable to sparse data and entails acquiring under-sampled datasets (i.e. fewer angular projections) and reconstructing the 3-D image stacks using an iterative algorithm. Here we use the TwIST algorithm [25], which we have implemented in MATLAB (The MathWorks Inc) and which requires approximately 30 minutes on a multicore personal computer (with Intel®Xeon® E5-2630 processor, 128 GB RAM and NVIDIA Tesla K40 GPU) to reconstruct a 3-D image stack using 64 angular projections. This iterative reconstruction process followed that described in reference [14] and the same default values were used for all parameters excluding the regularisation parameter, τ, and the number of total variation minimisations, TV_it_. For the tumour reconstructions, τ was set to 0.015 with TV_it_ set to 25 and, for the vasculature reconstructions, τ was set to 0.004 and TV_it_ set to 10. These values were selected based upon a compromise between reconstruction quality and time.

### Image segmentation and quantification

A percentile threshold based upon the cumulative frequency graph was applied to both tumour and vasculature reconstructions. For quantitative analysis of the vasculature, a median filter was applied over a neighborhood of 5 pixels, before using a multiscale Hessian-based method [26] in MATLAB for vessel enhancement. This determines the “vesselness”, i.e. the likelihood of each voxel belonging to a vessel, by analysing local image structure in the form of Hessian eigenvalues. The eigenvalues represent variations in intensity in three orthogonal directions and it is deemed likely the voxel belongs to a vessel if one eigenvalue is small and the other two are of large magnitude and negative, considering that the vessel structures are brighter than the background. The input parameters used were as previously described [14].

Following the iterative reconstruction of the 3-D data for the cross-sectional and longitudinal studies, the resulting “z-stacks” could be analysed to quantify the eGFP-labelled tumour volume and the changes in the structure of the mCherry-labelled vasculature, respectively. The tumour volume was quantified through extracting the number of non-zero voxels in the image. For the vasculature, the output of the Hessian-based analysis was subjected to a threshold before being imported into the Amira (FEI) software package. In Amira a 3-D median filter with a neighborhood of 6 was used before applying the auto-skeleton tool with no threshold, 10 iterations and “smooth” and “attach to data” coefficients of 0.5 and 0.25, respectively. From the resultant skeleton vessel length, volume, diameter and branching were analysed for the tumour region. Vessels with diameter smaller than 26 μm were excluded from the analysis. For visualisation the final CS-OPT images are presented with the skeletonized and dilated vasculature to present a binary image with appropriate vessel diameters. In the final segmented images, vasculature extending up to ~1.7 mm into fish has been reconstructed.

### Histopathology and Immunohistochemistry

For the cross-sectional study of HCC progression, whole fish were fixed in 10% formalin (Fischer Scientific, P/0840/53) following imaging for subsequent tumour dissection and histological analysis. Dissected tumours were paraffin embedded and sagittal sectioned at 5 μm. Histopathological analysis of tumour progression was performed on haematoxylin- and eosin-stained sections digitally imaged using a NanoZoomer (Hamamatsu, UK). Immunohistochemistry (IHC) was performed by IQPath: (UCL- https://www.ucl.ac.uk/ion/divisions/neuropathology/ion-histology/histology). Staining was performed using the Ventana Discovery XT instrument, using the Ventana DAB Map detection kit (760-124). Heat mediated epitope demasking was performed on the Ventana instruments, using a citrate based buffer (Ventana Ribo CC, 760-107). Anti-mCherry (Novus Biologicals, 1C51) primary antibody incubation was for 8 hours using a 1:100 dilution. Rabbit anti- Mouse (Dako E0354) secondary antibody incubation was for 32 minutes, using a 1:200 dilution. Slides were haematoxylin counterstained. Digital images of the stained sections were generated using a Leica SCN400F at x40 magnification.

### Immunohistochemistry Quantification

Analysis of the digital IHC images was performed at x20 magnification. Manually selected regions of interest (ROI) corresponding to segmented vasculature in the CS-OPT data were analysed using Definiens Developer XD v2.4.2 (Munich, Germany). Initial identification of the tissue, background and vessels within these ROI was based on the level of blue and brown staining, calculated from the RGB image using HSD model [27]. The combined level of brown and blue staining was used to identify the tissue and background; background ≤ 0.5 > Tissue (value range 0-3 a.u.). The threshold used to identify vessels (Th_V) was calculated using percentiles decreasing from 99 in increments of 1, searching for a change in value greater than 0.01au; a percentile of 99 describes the threshold that separates the lowest stained 99% of tissue from the highest 1%. A lower threshold (Th_L) was then calculated at Th_V-4. Following identification of all areas with brown staining greater than Th_V, any object with blue staining equal to or greater than the level of brown staining was removed. The remaining brown areas were then grown into surrounding pixels with brown staining greater than Th_L. Vessel lumen were then incorporated and vessel diameters calculated. Percent tumour vascularisation was based on the vessel stain plus lumen for the area of tissue plus enclosed background. Due to changes in vessel morphology upon tissue fixation (i.e. collapsed vessels) and limits of CS-OPT resolution as described above, vessels with diameter greater than 100 μm or lower than 5 μm and diameter lower than 10μm with a width/length ratio over 3 were excluded from analysis.

### Statistical analysis

Statistical analysis was performed by one-way analysis of variance (ANOVA), where *p* <0.05 was considered significant.

## SUPPLEMENTARY FIGURES AND VIDEOS










