# Supplementary material for: Quantitative in vivo optical tomography of cancer progression & vasculature development in adult zebrafish
Source: Oncotarget. 2016 Jun 1;7(28):43939–48. doi: 10.18632/oncotarget.9756 (PMC5190069; doi:10.18632/oncotarget.9756)
Supplement: Supplementary file 1 [file oncotarget-07-43939-s001.pdf]

Quantitative *in vivo* optical tomography of cancer progression & vasculature development in adult zebrafish

SUPPLEMENTARY FIGURES AND VIDEOS

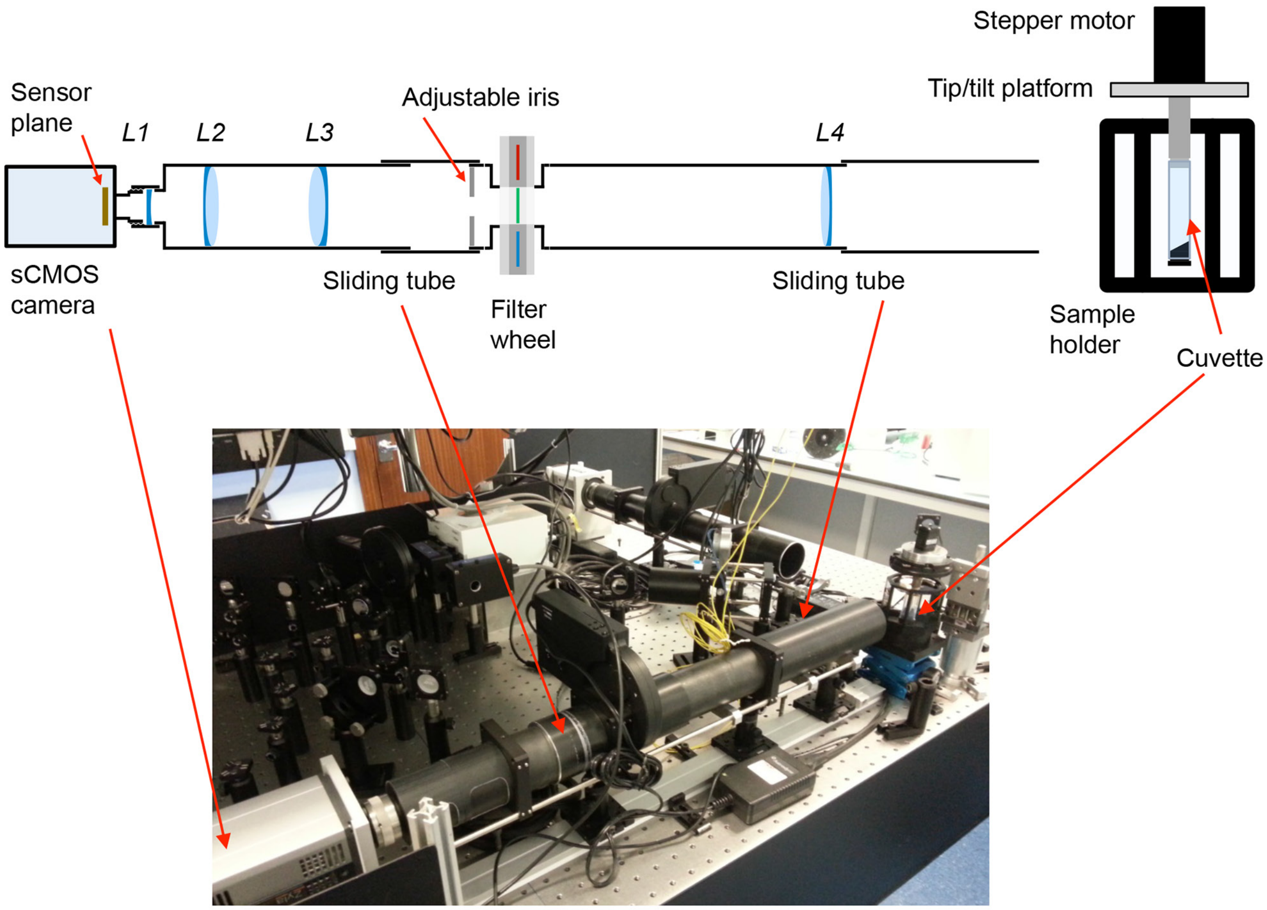

Supplementary Figure S1: Schematic of imaging arm for acquiring projection images with photograph of OPT set-up. The lenses are available from Thorlabs, Inc. (L1: LF1988-A [f=-500 1” singlet meniscus]; L2: AC504-200-A [f=200 2” achromatic doublet], L3: AC508-180-A [f=180 2” achromatic doublet]; L4: AC508-250-A [f=250 2” achromatic doublet]).

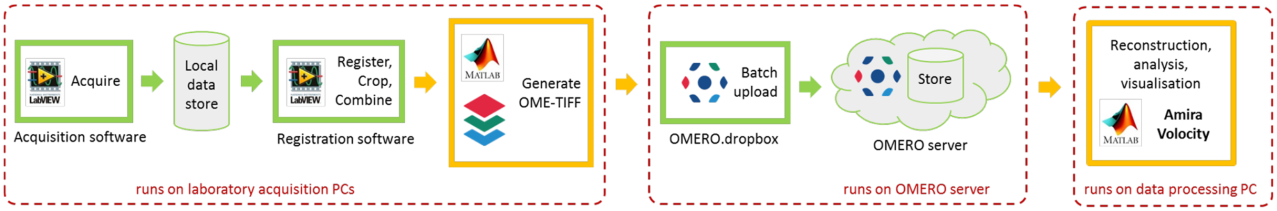

Supplementary Figure S2: Schematic of OPT data acquisition, registration, storage and reconstruction workflow.

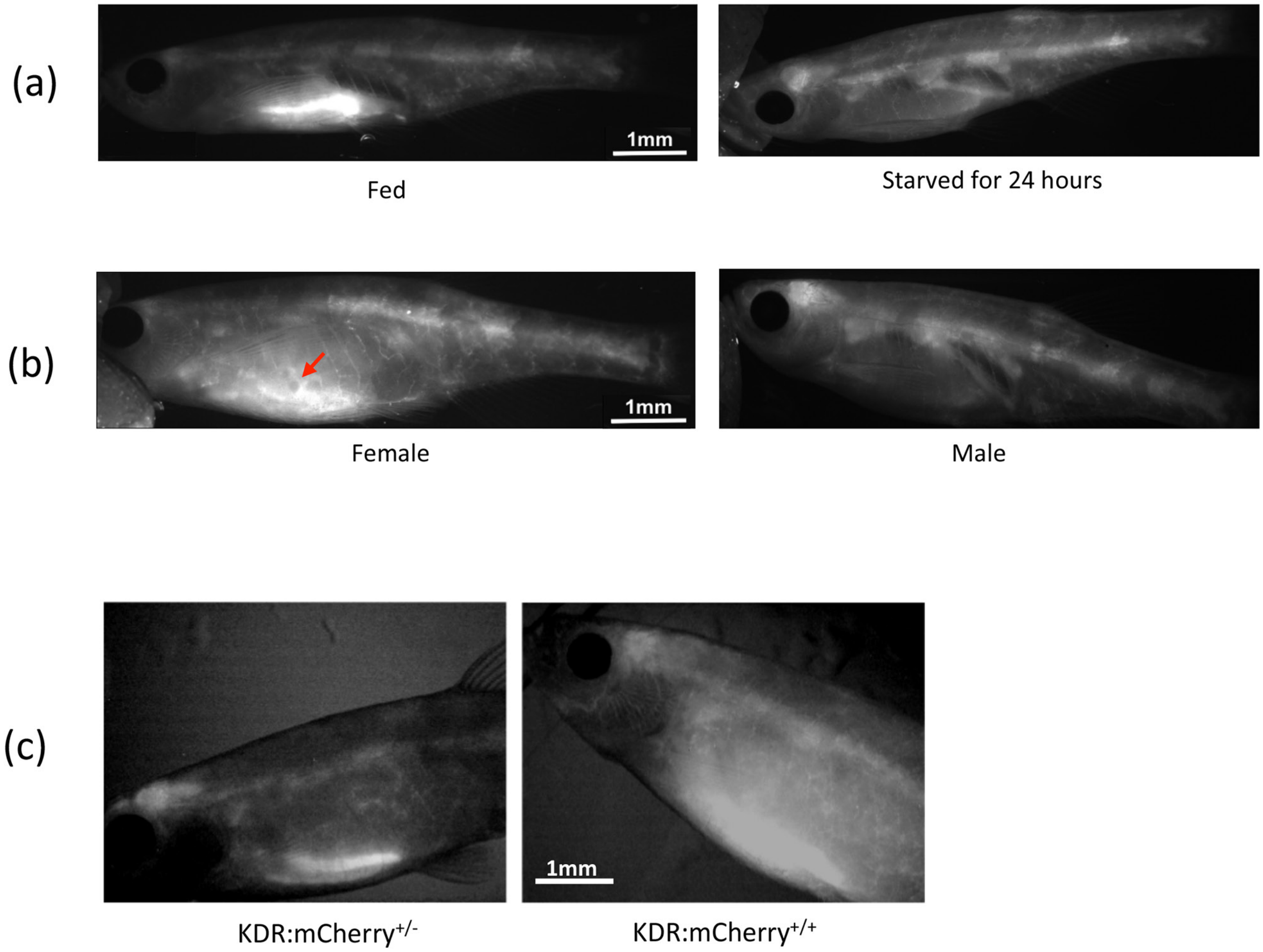

Supplementary Figure S3: Fluorescent properties of adult TraNac *Tg* (*KDR:mCherry:Fabp10-rtTA:TRE-eGFPKRAS*^{V12}) zebrafish excited at 561nm. a. Wide-field fluorescence intensity images (acquired as individual OPT projections) of fed and starved zebrafish indicating auto fluorescence in the gut of fed zebrafish. **b.** Wide-field fluorescence intensity images of starved zebrafish indicating light scattering in abdomen due to eggs in female (arrow indicates scattering by the eggs). **c.** Wide field fluorescence microscope images of heterozygous (KDR:mCherry$^{(+/-)}$) and homozygous (KDR:mCherry$^{(+/+)}$) zebrafish.

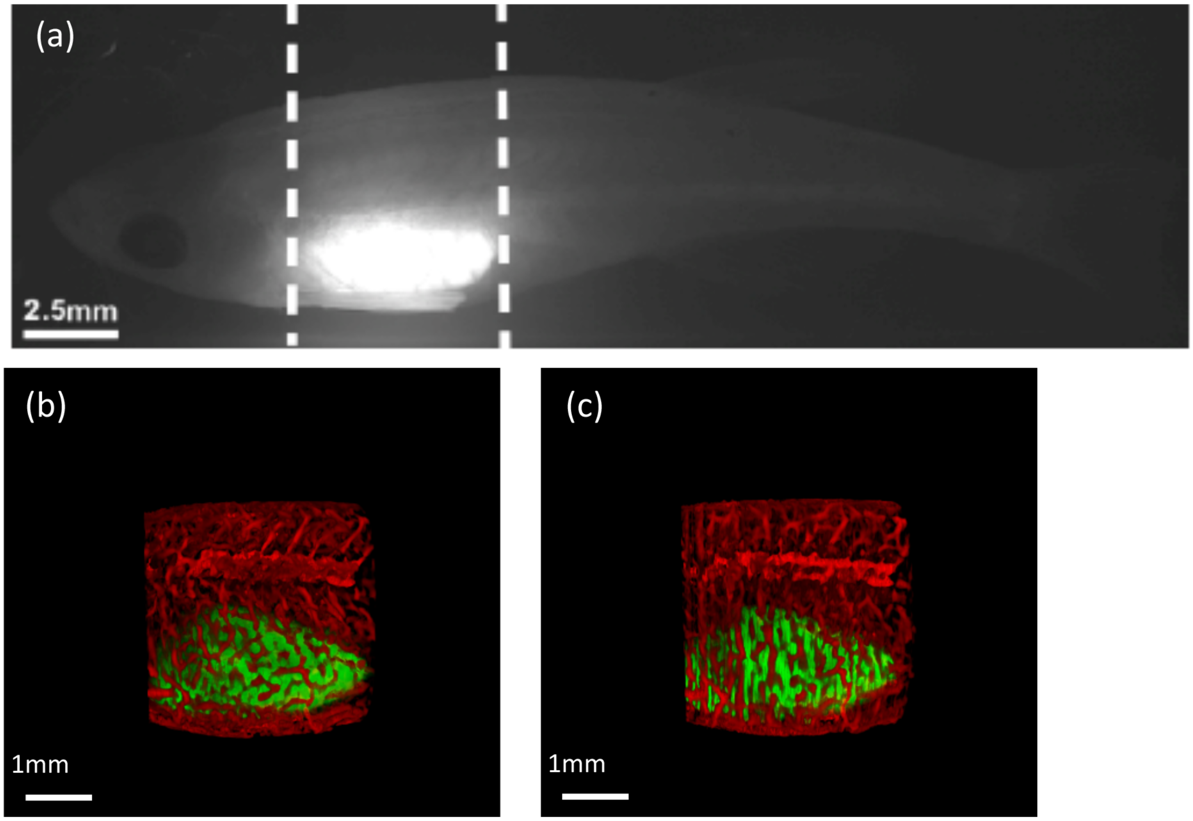

Supplementary Figure S4: 3-dimensional reconstructions of adult TraNac *Tg* (*KDR:mCherry:Fabp10-rtTA:TRE-eGFPKRAS*^{V12}) zebrafish after tumour induction using FBP and CS. a. Wide-field fluorescence intensity image (acquired as individual OPT projection) of tumour excited at 488 nm. **b, c.** Reconstructions of the tumour and vasculature (Hessian-based analysis) in the region indicated by dotted lines using (b) FBP with 512 Projections and (c) CS with 64 projections respectively.

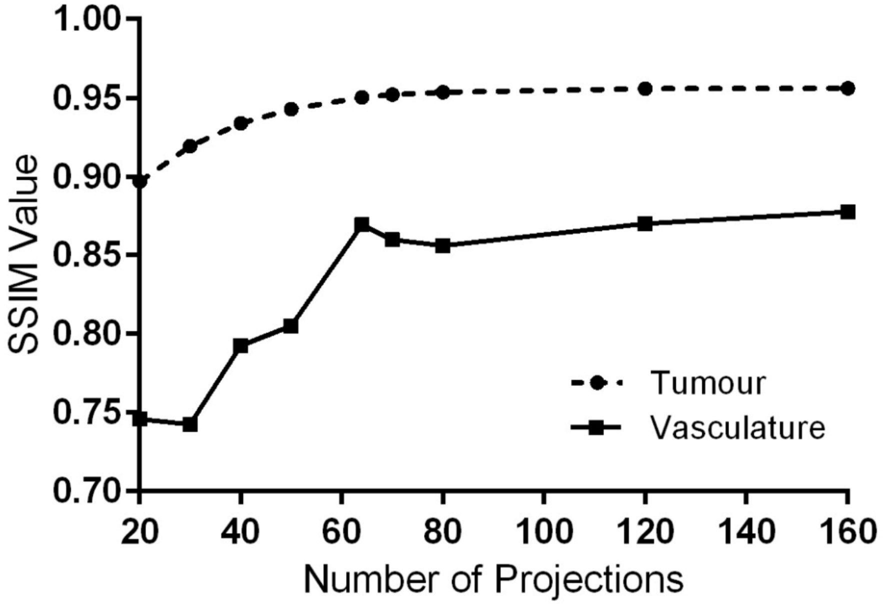

Supplementary Figure S5: Quantitative analysis of fidelity of reconstructed images using CS showing structural similarity index measure (SSIM) as a function of the number of angular projections for tumour and vasculature channels.

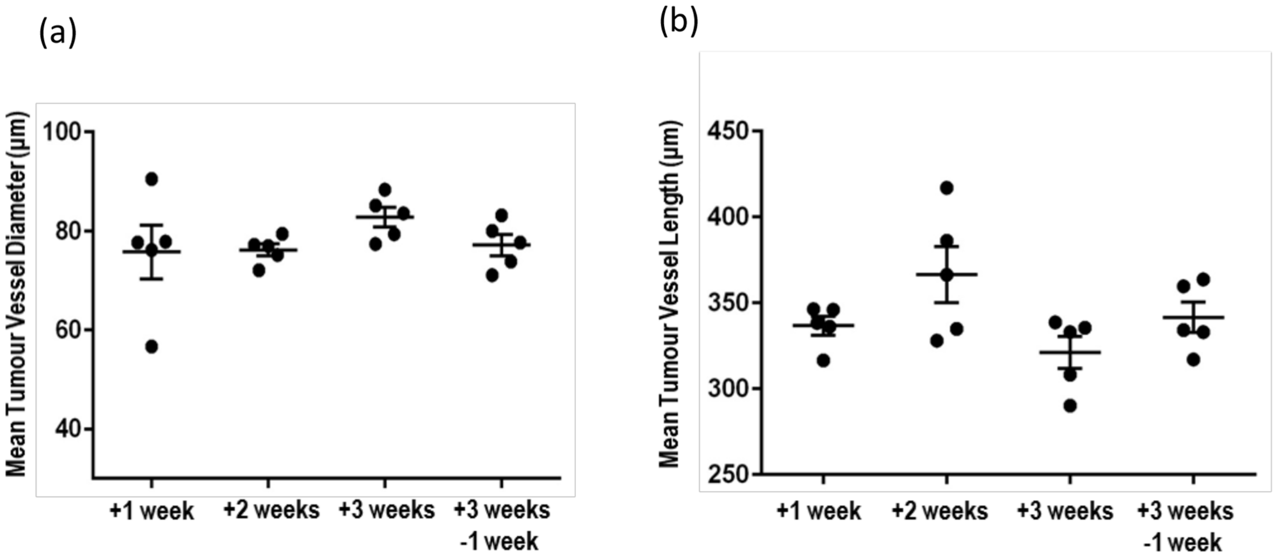

Supplementary Figure S6: Quantitative analysis of vasculature in "final" CS-OPT images from the cross-sectional study of adult (145 dpf) TraNac *Tg (KDR:mCherry;Fabp10:rtTA;TRE:eGFP:KRAS^{V12})* zebrafish following one, two and three weeks of DOX treatment and after three weeks of DOX treatment plus a further week with no treatment. a. shows the mean tumour vessel diameter and **b.** the mean tumour vessel length, for which n=5 at each condition and the error bars represent the standard error of the mean (SEM). *p<0.05, **p<0.01 as indicated by lines.

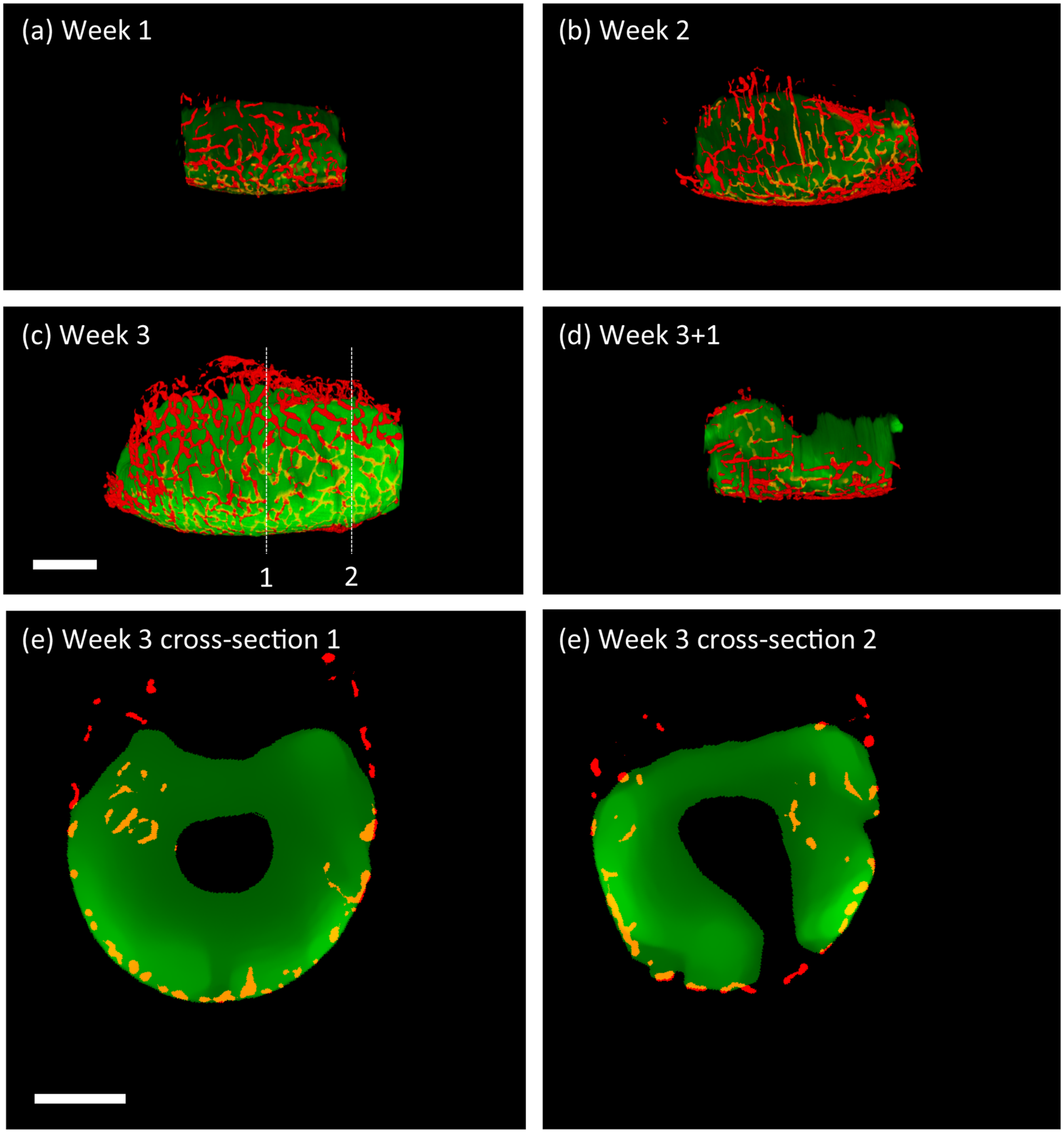

Supplementary Figure S7: a-d. Representative "final" segmented reconstructed CS-OPT images of eGFP-labelled tumour and mCherry-labelled vasculature in adult TraNac Tg (KDR:mCherry;Fabp10:rtTA;TRE:eGFP:KRASV12) zebrafish with data acquired following DOX treatment at 145 dpf after (a) one, (b) two, (c) three weeks of treatment and (d) after three weeks of DOX treatment plus a further week with no treatment (See also Supplementary Video 3). **e, f.** Representative cross-sections showing tumour and vasculature from the reconstructed volume shown in (c) indicated by dotted lines 1 and 2 respectively. Red – vasculature 'outside' tumour, orange – vascular 'inside' tumour, green - tumour. Scale bar 2 mm.

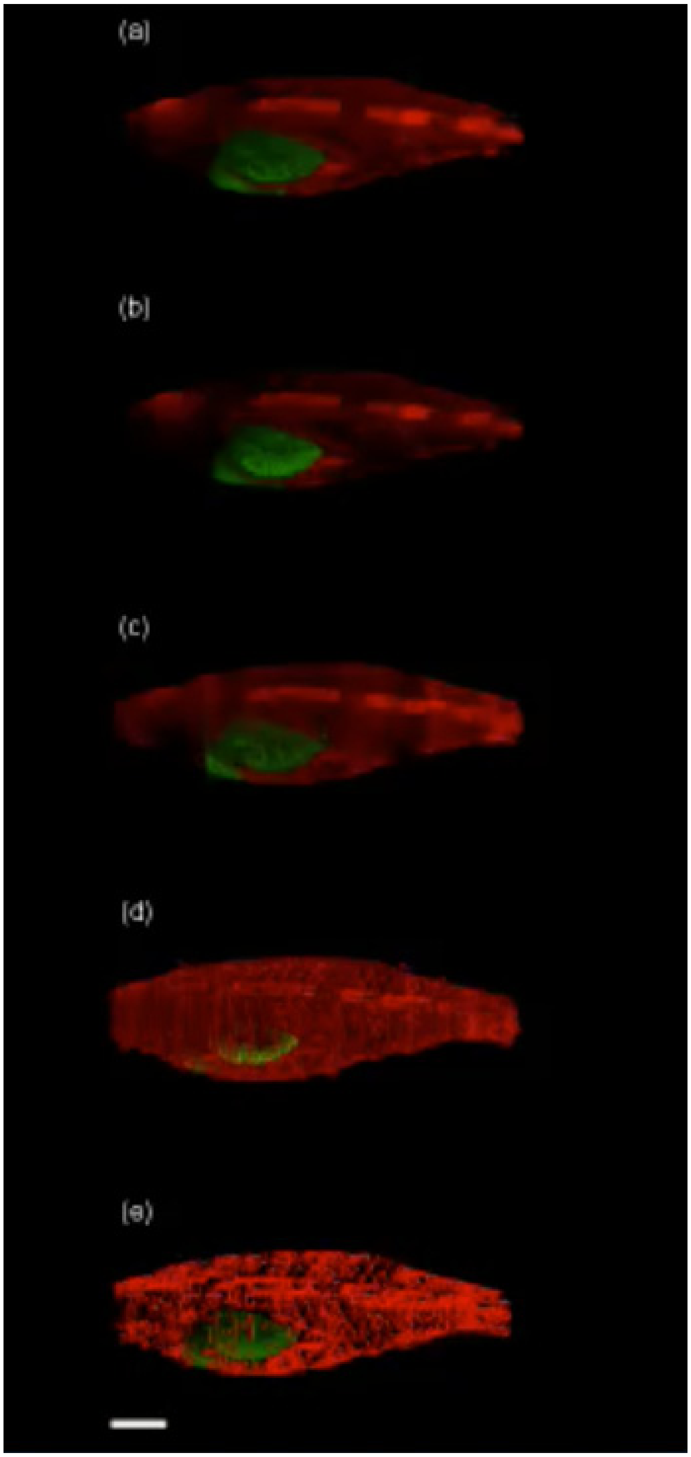

Supplementary Video 1: Movie of exemplar reconstructed OPT images of adult (TraNac *Tg* (*KDR:mCherry:Fabp10-rtTA:TRE-eGFPKRAS^{V12}*)) zebrafish expressing liver specific GFP-labelled tumour and mCherry-labelled vasculature acquired with 2-axis OPT system, showing maximum intensity projection of GFP (green) and mCherry (red) fluorescence. **a, b.** show rendered 3-D FBP reconstructions computed with 512 and 64 projections respectively, **c.** shows a CS-OPT reconstruction from 64 projections using the TwIST algorithm, **d.** shows the "vesselness" of (c) after Hessian-based analysis and **e.** shows the "final" segmented CS-OPT reconstruction with segmented vasculature following skeletonization and dilation. Scale bar 2.5 mm. Each group (n=8).

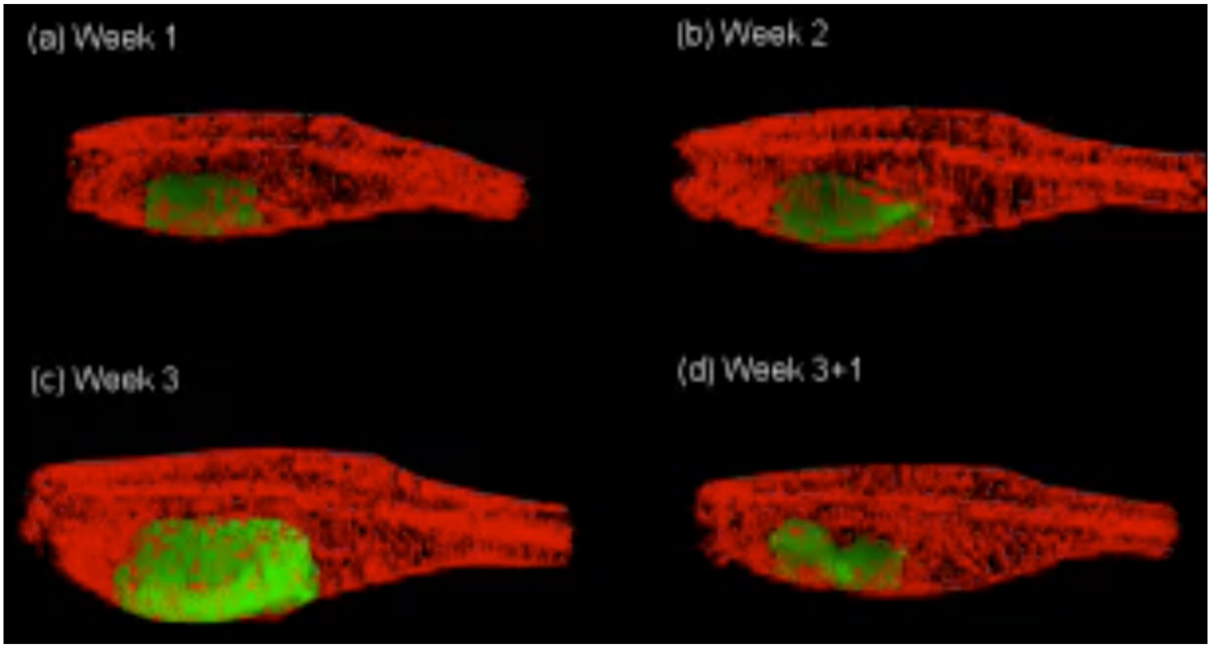

Supplementary Video 2: Representative "final" reconstructed CS-OPT movies of adult TraNac *Tg* **(***KDR:mCherry:Fabp10-rtTA:TRE-eGFPKRAS*^{V12}) zebrafish following tumour induction for a. one week, **b.** two weeks and **c.** three weeks with **d.** imaged after three weeks of induction plus a further week after removal of inducer treatment. Scale bar 5 mm. Each group (n=8).

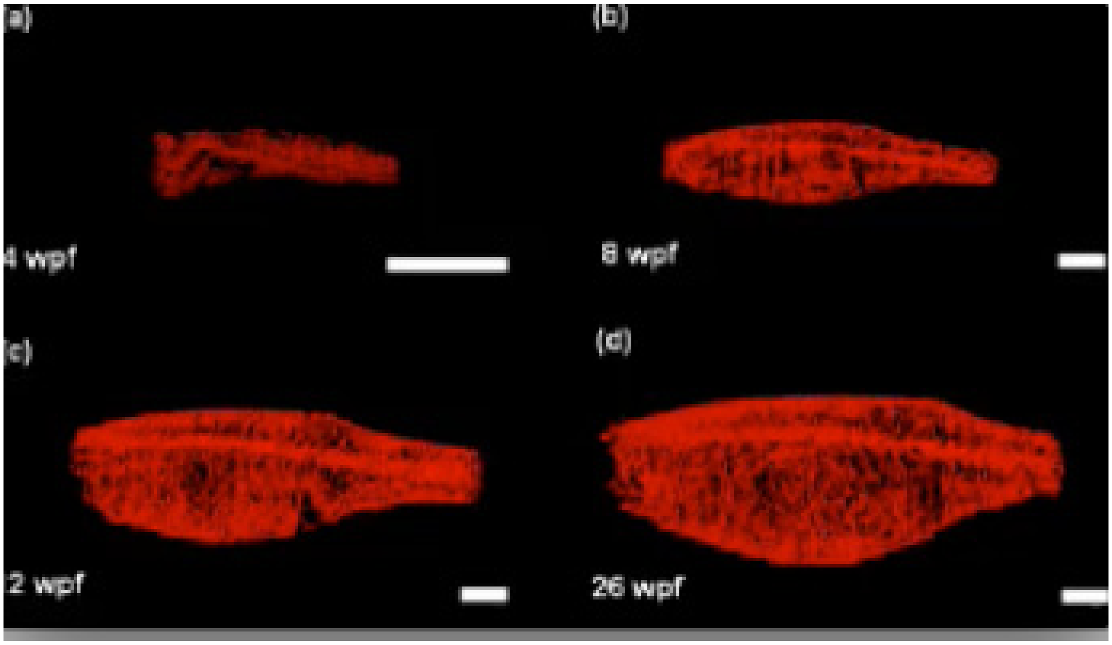

Supplementary Video 3: Representative "final" reconstructed CS-OPT movies of cropped tumour regions from adult TraNac Tg (KDR:mCherry:Fabp10-rtTA:TRE-eGFPKRASV12) zebrafish following tumour induction for **a.** one, **b.** two and **c.** three weeks with **d.** imaged after three weeks of induction plus a further week after removal of inducer treatment. **e.** Fly-through movie showing reconstructed cross-sectional images for volume shown in (c). Red – vasculature 'outside' tumour, orange – vascular 'inside' tumour, green - tumour. Scale bar 2 mm.

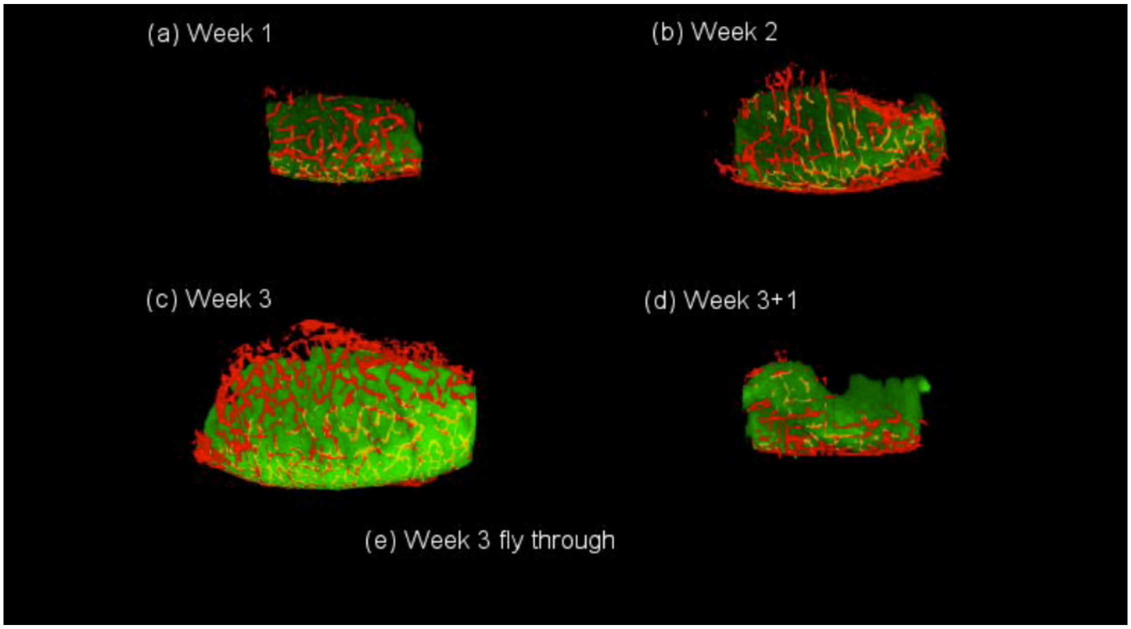

Supplementary Video 4: Representative "final" reconstructed CS-OPT movies corresponding to Figure 2g showing reconstructed images from the CS-OPT data of TraNac *Tg (KDR:mCherry)* zebrafish imaged in longitudinal study over 26 weeks showing images acquired at **a.** 4, **b.** 8, **c.** 12 and **d.** 26 weeks post fertilization. Scale bar 2 mm.
